# Use of Multiplex Quantitative PCR To Evaluate the Impact of Pneumococcal Conjugate Vaccine on Nasopharyngeal Pneumococcal Colonization in African Children

**DOI:** 10.1128/mSphere.00404-17

**Published:** 2017-11-08

**Authors:** Courtney P. Olwagen, Peter V. Adrian, Marta C. Nunes, Michelle J. Groome, Mark F. Cotton, Avy Violari, Shabir A. Madhi

**Affiliations:** a Department of Science and Technology/National Research Foundation, Vaccine Preventable Diseases, Faculty of Health Sciences, University of the Witwatersrand, Johannesburg, South Africa; b Medical Research Council: Respiratory and Meningeal Pathogens Research Unit, University of the Witwatersrand, Johannesburg, South Africa; c Children’s Infectious Diseases Clinical Research Unit, Stellenbosch University, Tygerberg, South Africa; d Perinatal HIV Research Unit, University of the Witwatersrand, Johannesburg, South Africa; U.S. Food and Drug Administration

**Keywords:** PCV7 vaccination, serotype replacement, unmasking

## Abstract

Pneumococcal conjugate vaccine (PCV) immunization of children induces shifts in colonizing pneumococcal serotypes. This study evaluated the effect of infant vaccination with 7-valent PCV (PCV7) on vaccine serotype (VT) colonization and whether the increase in nonvaccine serotype (NVT) was due to either unmasking of previously low-density-colonizing serotypes or increase in acquisition of NVT. A multiplex quantitative PCR (qPCR) was used to evaluate VT and NVT nasopharyngeal colonization in archived swabs of PCV-vaccinated and PCV-unvaccinated African children at 9 and 15 to 16 months of age. Molecular qPCR clearly identified the vaccine effect typified by a decrease in VT colonization and an increase in NVT colonization. Serotype 19A was primarily responsible for the higher NVT carriage among PCV vaccinees at 9 months of age (53.4% difference; *P* = 0.021) and 16 months of age (70.7% difference; *P* < 0.001). Furthermore, the density of serotype 19A colonization was higher in PCV-vaccinated groups than in PCV-unvaccinated groups (3.76 versus 2.83 CFU/ml [*P* = 0.046], respectively, and 4.15 versus 3.04 CFU/ml [*P* = 0.013], respectively) at 9 and 16 months of age, respectively. Furthermore, serotype 19A was also more commonly reported as a primary isolate (by having the highest density among other cocolonizing serotypes identified in the sample) in PCV7-vaccinated children, while being equally a primary (46.2%) or nonprimary (53.8%) isolate in PCV-unvaccinated children. Molecular qPCR showed both serotype replacement and unmasking to be the cause for the increase in NVT colonization in PCV7-vaccinated children, as some serotypes were associated with an absolute increase in colonization (replacement), while others were associated with an increase in detection (unmasking).

**IMPORTANCE** This study focused on evaluating the effect of infant vaccination with 7-valent pneumococcal conjugate vaccine (PCV7), using a multiplex qPCR method, on the density of serotype-specific nasopharyngeal colonization in order to delineate the relative role of serotype replacement versus unmasking as the cause for the increase in nonvaccine serotype colonization in PCV7-vaccinated children. This is pertinent in the context of the ongoing deployment of PCV immunization in children, with surveillance of colonization considered an early proxy for disease that might arise from nonvaccine serotypes, as well as the success of childhood vaccination on indirect effect in the community through the interruption of pneumococcal transmission from vaccinated young children.

## Abstract (precis)

This study focused on evaluating the effect of infant vaccination with 7-valent pneumococcal conjugate vaccine (PCV7), using a multiplex qPCR method, on the density of serotype-specific nasopharyngeal colonization in order to delineate the relative role of serotype replacement versus unmasking as the cause for the increase in nonvaccine serotype colonization in PCV7-vaccinated children. This is pertinent in the context of the ongoing deployment of PCV immunization in children, with surveillance of colonization considered an early proxy for disease that might arise from nonvaccine serotypes, as well as the success of childhood vaccination on indirect effect in the community through the interruption of pneumococcal transmission from vaccinated young children.

## INTRODUCTION

The human nasopharynx is colonized with multiple commensal and some potentially pathogenic organisms, including *Streptococcus pneumoniae* (1). Vaccination of children with pneumococcal conjugate vaccines (PCV) reduces the risk of *S. pneumoniae* vaccine serotype (VT) nasopharyngeal colonization but is associated with increased detection of nonvaccine serotypes (NVT) (2). Increased NVT colonization in vaccinees could involve either replacement colonization through reduced VT acquisition or unmasking of previously prevailing NVT that were not identified by traditional culture methods that detect only the dominant colonizing serotype or both mechanisms (3). Molecular detection of pneumococci in the nasopharynx has several advantages over traditional culture-based methods, including the detection of multiple serotypes from a single sample with high sensitivity, as well as quantitative PCR (qPCR) methods being able to measure the density of colonization and relative proportion of colonizing serotypes (4).

The study aimed to use qPCR to evaluate the effect of infant vaccination with 7-valent PCV (PCV7) on the density of serotype-specific nasopharyngeal colonization to delineate the relative roles of serotype replacement and unmasking to explain the increase in NVT colonization in PCV7-vaccinated children.

## RESULTS

**FIG 1:**
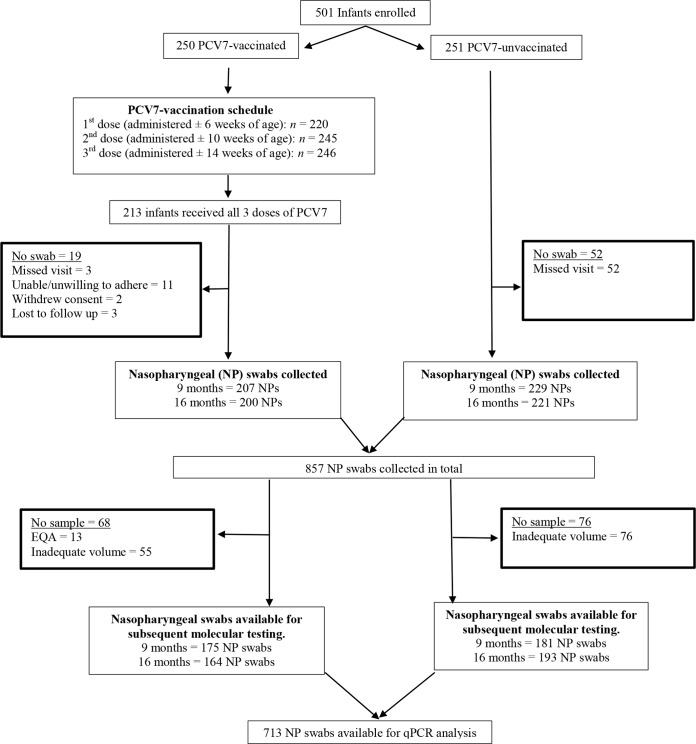
Schematic diagram of study population. Flow diagram indicating the number of children initially enrolled in a PCV-unvaccinated and PCV7-vaccinated cohort of HIV-uninfected children, as well as the number of nasopharyngeal (NP) swabs available for subsequent quantitative PCR (qPCR) analysis. PCV7-vaccinated participants were excluded from analysis if they did not receive all three doses of the pneumococcal conjugate vaccine (PCV) within protocol-defined window periods. The number of NP swabs available for molecular testing was defined by whether there was an adequate volume of sample remaining. Some samples were used for external quality assessment (EQA).

Quantitative PCR analysis involved 713 (83%) of the initial 857 nasopharyngeal swabs collected from children at either 9 or 16 months of age (Fig. 1). Among the children with samples available for testing, 58% were HIV exposed and 52% were male (Table 1). In the PCV7-vaccinated group, a lower percentage of children were black African compared to the PCV-unvaccinated group (83% versus 100%; *P* < 0.001). The percentage of children attending day care and having a smoking household contact was higher in PCV7-vaccinated children at 9 months of age (day care, 52% versus 22.1% [*P* < 0.001]; smoking contact, 42.3% versus 36.5% [*P* = 0.003]) and 16 months of age (day care, 53.6% versus 37.3% [*P* = 0.002]; smoking contact, 39% versus 33.7% [*P* = 0.004]), while the mean age (in months) at the time of sample collection was 9 (standard deviation [SD], 0.7) and 15.4 (SD, 0.4) in PCV7-vaccinated children and 9.4 (SD, 0.5; *P* < 0.001) and 16 (SD, 1.4; *P* < 0.001) in PCV-unvaccinated children. Although the differences in age were small and unlikely to be of epidemiological or clinical significance, we chose to adjust the subsequent analyses for age in addition to the other variables that were different between the two cohorts.

**TABLE 1:** Demographic features of PCV-unvaccinated and PCV7-vaccinated, HIV-uninfected children at two study visits when nasopharyngeal bacterial colonization was analyzed^a^

Demographic feature^b^	Value^b^ for 9-mo-old children		*P* value	Value for 16-mo-old children		*P* value
	PCV-unvaccinated	PCV7-vaccinated		PCV-unvaccinated	PCV7-vaccinated	
No. of children enrolled	250	251		250	251	
No. of NP swabs available for molecular analysis	181	175		193	164	
HIV status						
Exposed, *n* (%)	111 (61.3)	104 (59.4)	0.40	99 (51.3)	97 (59.1)	0.24
Unexposed, *n* (%)	70 (38.7)	71 (40.6)		94 (48.7)	67 (40.9)	
Birth wt (g), mean (SD)	3,061 (440)	3,097 (482)	0.45	3,079 (427)	3,126 (476)	0.43
Male sex, *n* (%)	87 (48.1)	96 (54.9)	0.12	99 (51.3)	89 (54.3)	0.25
Race						
Black African, *n* (%)	181 (100)	150 (85.7)	<0.001	193 (100)	132 (80.5)	<0.001
Mixed ancestry, *n* (%)		25 (14.3)			32 (19.5)	
Smoking household contact, *n* (%)	66 (36.5)	81 (42.3)	0.003	65 (33.7)	64 (39)	0.004
Received co-trimoxazole prophylaxis, *n* (%)	95 (52.5)	81 (46.3)	0.2	71 (36.8)	68 (41.5)	0.15
No. of children <5 years old in the household, mean (SD)	1.6 (0.8)	1.6 (0.7)	0.6	1.6 (0.8)	1.5 (0.8)	0.21
No. of household contacts, mean (SD)	5.3 (2.2)	5.2 (2.3)	0.81	5.2 (2.1)	5.1 (2.4)	0.17
Day care attendance, *n* (%)	40 (22.1)	91 (52)	<0.001	72 (37.3)	81 (53.6)	0.002
Breastfeeding, *n* (%)	41 (22.7)	44 (25.1)	0.33	37 (19.2)	38 (23.3)	0.43
Age (mo) at visit, mean (SD)	9 (0.7)	9.4 (0.5)	<0.001	15.4 (0.4)	16 (1.4)	<0.001

a The Pearson χ^2^ test or Student *t* test was used to compare baseline characteristics between the two study cohorts, and demographic features with a *P* value of <0.2 were included as possible cofounders in multivariate analysis. PCV7, 7-valent pneumococcal conjugate vaccine.

b Values are the number of children (*n*) and percentage unless specified otherwise. NP, nasopharyngeal; SD, standard deviation.

### Prevalence of nasopharyngeal pneumococcal colonization in PCV-unvaccinated and PCV7-vaccinated children.

**FIG 2:**
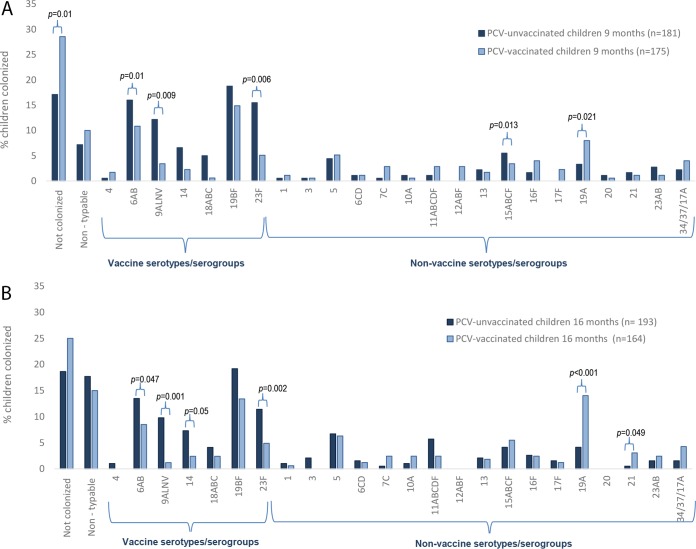
Prevalence of nasopharyngeal (NP) pneumococcal colonization in PCV7-vaccinated and PCV-unvaccinated, HIV-uninfected children who were 9 months old (A) and 16 months old (B). The *P* values were determined by multivariate logistic regression, controlling for race, smoking household contact, co-trimoxazole use, day care attendance, and mean age at the time of sample collection by using generalized estimating equations. *P* values of <0.05 were considered significant. PCV7, seven-valent pneumococcal conjugate vaccine.

When the children were 9 months old, overall pneumococcal colonization prevalence was lower in PCV7-vaccinated children (71%) than in PCV-unvaccinated children (82.9%; *P* = 0.01), which was due to lower colonization prevalence of PCV7 serotypes (36% versus 61.9%; *P* = 0.002) (Table 2). Specifically, PCV7 serogroup 9A/L/N/V (72.1% difference, *P* = 0.009) and 23F (67.1% difference; *P* = 0.006) (Fig. 2A). A corresponding higher colonization prevalence of NVT in PCV7-vaccinated (40%) compared to PCV-unvaccinated children (33.7%; *P* = 0.02) was evident at 9 months of age; largely driven by higher prevalence of a limited number of serotypes/serogroups, mainly serogroup 12A/B/F (100% difference; *P* = 0.013) and 19A (53.4% difference;, *P* = 0.021).

**TABLE 2:** Prevalence of pneumococcal nasopharyngeal colonization in PCV7-vaccinated and PCV-unvaccinated, HIV-uninfected children as measured by quantitative qPCR

Pneumococcus^a^	Prevalence in 9-mo-old children^b^		OR (95% CI), * P* value^c^	aOR^d^ (95% CI), *P* value	Prevalence in 16-mo-old children		OR (95% CI), *P* value	aOR (95% CI), *P* value
	PCV7- unvaccinated (*n* = 181)	PCV7- vaccinated (*n* = 175)			PCV7- unvaccinated (*n* = 193)	PCV7- vaccinated (*n* = 164)		
*lytA*	150 (82.9)	125 (71.4)	0.52 (0.31–0.86), *P* = 0.01	0.45 (0.23–0.87), *P* = 0.01	157 (81)	123 (75)	0.68 (0.41–1.14), *P* = 0.147	0.55 (0.29–1.03), *P* = 0.06
VT serotypes	112 (61.9)	63 (36)	0.35 (0.21–0.58), *P* < 0.001	0.37 (0.19–0.7), *P* = 0.002	100 (51.8)	54 (32.9)	0.44 (0.27–7.01), *P* < 0.001	0.41 (0.26–0.63), *P* = 0.007
NVT serotypes	54 (33.7)	70 (40)	1.74 (1.08–2.82), *P* = 0.002	1.88 (1.02–3.48), *P* = 0.02	73 (37.8)	102 (62.2)	1.81 (1.12–2.93), *P* < 0.001	2.2 (1.18–4.1), *P* = 0.01

a *Streptococcus pneumoniae* carrying the *lytA* gene or *S*. *pneumoniae* serotypes. PCV7 (7-valent pneumococcal conjugate vaccine) serotypes/serogroups (VT) include 4, 6A/B, 9A/L/N/V, 14, 18 A/B/C, 19B/F, and 23F. Nonvaccine serotypes/serogroups (NVT) are 1, 3, 5, 6 C/D, 7C, 10A, 11A/B/C/D/F, 12A/B/F, 13, 15 A/B/C/F, 16F, 17F, 19A, 20, 21, 23A/B, and 34/37/17A.

b Prevalence of pneumococcal nasopharyngeal colonization in 9-month-old or 16-month-old children vaccinated or not vaccinated with PCV7. The number of children colonized with pneumococcus is shown. The percentage of children colonized is shown in parentheses.

c The odds ratio (OR), the 95% confidence interval (95% CI) for the odds ratio (shown in parentheses), followed by the *P* value.

d The adjusted odds ratio (aOR) for pneumococcal colonization was determined by multivariate logistic regression, controlling for race, smoking household contact, co-trimoxazole use, day care attendance, and mean age at the time of sample collection.

By the time the children were 16 months old, the difference in overall pneumococcal colonization between PCV7-vaccinated children (75%) and PCV-unvaccinated children (81%; *P* = 0.06) was not significant. The lower PCV7 serotype colonization prevalence in PCV7-vaccinated (32.9%) than in PCV-unvaccinated children (51.8%; *P* = 0.007) was largely offset by the higher prevalence of NVT colonization among the PCV7-vaccinated children (62.2% versus 37.8%; *P* = 0.013) (Table 2). The serotypes/serogroups that were less prevalent among PCV7-vaccinated children compared to PCV-unvaccinated children were 9A/L/N/V (87.8% difference; *P* < 0.001), 14 (67.1% difference; *P* = 0.05), and 23F (57% difference; *P* = 0.02), while nonvaccine serotypes 19A (70.7% difference; *P* < 0.001) and 21 (83.3% difference; *P* = 0.049) were more prevalent among PCV7-vaccinated children (Fig. 2B).

### PCV vaccination and density of pneumococcal carriage.

When the children were 9 months old, pneumococcal colonization density was higher in PCV7-vaccinated children than in PCV-unvaccinated children (4.68 versus 4.28 CFU/ml; *P* = 0.007) (Table 3). This was associated with a higher density overall of PCV7 serogroups/serotypes (3.8 versus 3.4 CFU/ml; *P* = 0.048), despite lower prevalence thereof, and higher overall density of NVT (3.6 versus 3.1 CFU/ml; *P* = 0.018). No difference in density of individual PCV7 serotypes/serogroups was found between PCV-unvaccinated and PCV7-vaccinated groups. The density of nonvaccine serotype 19A was, however, higher in PCV7-vaccinated children than in PCV-unvaccinated children (3.76 versus 2.83 CFU/ml; *P* = 0.046), for which there was also a higher colonization prevalence in PCV7-vaccinated children. Further, the density of nonvaccine serogroup 23A/B was also higher in PCV7-vaccinated children than in PCV-unvaccinated children (4.48 versus 2.07 CFU/ml; *P* = 0.002), despite no difference in colonization prevalence.

**TABLE 3:** Density of pneumococcal nasopharyngeal carriage in PCV7-vaccinated and PCV7-unvaccinated, HIV-uninfected children as measured by quantitative PCR

Pneumococcus	GMD (95% CI)^a^ in 9-mo-old children		*P* value	GMD (95% CI) in 16-mo-old children		*P* value
	PCV7-unvaccinated (*n* = 181)	PCV7-vaccinated (*n* = 175)		PCV7-unvaccinated (*n* = 193)	PCV7-vaccinated (*n* = 164)	
Pneumococcus (LytA)	4.28 (4.09 to 4.47)	4.68 (4.45 to 4.90)	0.007	4.33 (4.16 to 4.5)	4.44 (4.23 to 4.66)	0.53

Vaccine serotypes/ serogroups	3.4 (3.2 to 3.6)	3.8 (3.5 to 4.1)	0.048	3.59 (3.36 to 3.83)	3.73 (3.4 to 4.0)	0.48
4	3.8	2.18 (−2.1 to 6.47)		4.53	–	
6A/B	4.41 (4.0 to 4.82)	4.41 (3.9 to 4.92)	0.99	4.17 (3.66 to 4.68)	4.0 (3.44 to 4.58)	0.68
9A/L/N/V	2.52 (1.96 to 3.08)	2.84 (1.48 to 4.19)	0.52	2.36 (1.77 to 2.96)	3.99 (0.81 to 6.04)	0.09
14	2.46 (1.7 to 3.22)	3.66 (−0.68 to 6.65)	0.15	3.29 (2.68 to 3.91)	2.75 (1.29 to 4.20)	0.37
18A/B/C	2.80 (2.26 to 3.49)	1.96		3.58 (2.51 to 4.65)	4.30 (3.36 to 5.25)	0.32
19B/F	3.36 (2.99 to 3.74)	3.79 (3.28 to 4.3)	0.16	3.52 (3.12 to 3.96)	3.65 (3.12 to 4.17)	0.71
23F	2.9 (2.59 to 3.4)	3.76 (2.24 to 5.28)	0.84	3.04 (2.67 to 3.4)	3.44 (2.45 to 4.44)	0.71

Nonvaccine serotypes/ serogroups	3.1 (2.7 to 3.4)	3.6 (3.0 to 3.4)	0.018	3.33 (3.0 to 3.66)	3.74 (3.42 to 4.06)	0.08
1	2.31	2.29 (−1.03 to 5.61)		3.47 (−11.4 to 8.31)	1.99	
3	5.13	3.55		–	–	
5	2.0 (1.63 to 2.36)	2.29 (1.85 to 2.7)	0.23	2.01 (1.83 to 2.19)	2.79 (1.53 to 4.04)	0.015
6C/D	4.2 (−25.5 to 33.94)	2.76 (1.71 to 3.81)	0.60	2.99 (2.32 to 3.67)	3.99 (−12.12 to 0.1)	0.38
7C	3.25	3.13 (0.5 to 5.76)		1.57	2.66 (1.51 to 3.79)	
10B	4.15 (−10.47 to 8.78)	3.09		4.65 (4.0 to 5.3)	3.26 (2.15 to 4.36)	0.06
11A/B/C/D/F	1.79 (−2.66 to 6.24)	2.67 (1.32 to 4.0)	0.34	2.88 (1.9 to 3.87)	4.06 (0.82 to 7.3)	0.23
12A/B/F	2.44	3.38 (1.39 to 5.38)		–	–	
13	2.99 (0.22 to 6.01)	4.7 (2.86 to 6.54)	0.21	2.84 (1.08 to 4.61)	3.21 (1.38 to 5.04)	0.65
15A/B/C/F	3.66 (3.11 to 4.2)	4.1 (3.4 to 4.79)	0.27	3.88 (3.23 to 4.53)	4.03 (3.27 to 4.79)	0.75
16F	3.26 (2.24 to 4.28)	3.65 (2.4 to 4.89)	0.65	4.84 (2.67 to 7.01)	5.07 (2.77 to 7.37)	0.84
17F		–		3.10 (−1.31 to 7.5)	2.48 (−12.1 to 7.05)	0.72
19A	2.83 (2.26 to 3.7)	3.76 (3.36 to 4.1)	0.046	3.04 (2.26 to 3.83)	4.15 (3.66 to 4.64)	0.013
20	4.20 (2.33 to 6.07)	2.01		–	–	
21	3.62 (−0.65 to 7.89)	3.32 (−13.7 to 0.36)	0.87	5.22	2.82 (1.82 to 3.8)	
23A/B	2.07 (1.48 to 2.67)	4.48 (0.82 to 8.14)	0.002	1.58 (1.28 to 1.87)	3.59 (0.88 to 6.32)	0.013
34/37/17A	2.83 (1.33 to 4.34)	3.76 (2.24 to 5.28)	0.84	4.34 (0.50 to 8.17)	4.67 (3.7 to 5.61)	0.71

a The geometric mean density (GMD) of carriage and 95% confidence intervals (95% CI) were calculated following log_10_ transformations and compared with multivariate analysis controlling for race, smoking household contact, co-trimoxazole use, day care attendance, and mean age at time of sample collection. PCV7, 7-valent pneumococcal conjugate vaccine. −, too few observations to calculate the *P* value.

By the time the children were 16 months old, there was no overall difference in pneumococcal colonization density between the two study groups, although there was a higher density of NVT in PCV7-vaccinated children than in PCV-unvaccinated children, including serotypes/serogroups 5 (2.79 versus 2.01 CFU/ml; *P* = 0.015), 19A (4.15 versus 3.04 CFU/ml; *P* = 0.013), and 23A/B (3.59 versus 1.58 CFU/ml; *P* = 0.013), although there was only a higher prevalence of serotype 19A colonization

### Cocarriage of multiple serotypes.

Overall, qPCR detected one, two, and three or more serotypes in 42.8%, 16.5%, and 5.3% nasopharyngeal swabs, respectively. Additionally, 13.2% of the swabs were *lytA* positive but negative for all tested serotypes, implying the presence of nontypeable pneumococci, pneumococci belonging to untested serotypes, or *lytA*-positive nonpneumococcal streptococcal species. Colonized PCV7-vaccinated children were less likely to have only a single serotype identified than PCV-unvaccinated children at 9 months (59.2% versus 64.6%; *P* = 0.025) and 16 months (44.7% versus 50.3%; *P* = 0.019) of age (Table 4).

**TABLE 4:** Cocolonization by vaccine types and nonvaccine type pneumococcus in HIV-uninfected children as measured by quantitative molecular PCR

Pneumococcal colonization^a^	Cocolonization^b^ in 9-mo-old children		*P* value^c^	Cocolonization in 16-mo-old children		*P* value
	PCV7-unvaccinated (*n* = 181)	PCV7-vaccinated (*n* = 175)		PCV7-unvaccinated (*n* = 193)	PCV7-vaccinated (*n* = 164)	
Total	150 (82.9)	125 (71.4)	0.01	157 (81.3)	123 (75)	0.06
Not typeable^d^	14 (9.3)	22 (17.6)	0.09	29 (18.5)	29 (23.4)	0.88
Single carriers	97 (64.6)	74 (59.2)	0.025	79 (50.3)	55 (44.7)	0.019
Multiple carriers	39 (26)	29 (23.2)	0.87	49 (31.2)	39 (31.7)	0.63
+1 VT	12 (30.7)	4 (13.8)	0.002	11 (22.4)	1 (2.6)	0.005
VT and NVT	26 (66.7)	18 (62.1)	0.006	31 (63.2)	21 (55.3)	0.004
+1 NVT	1 (2.6)	7 (24.1)	0.015	7 (14.3)	16 (42)	0.002

a The total colonizing pneumococci isolated, including nontypeable pneumococci, single carriers, and multiple carriers (carrying more than one VT, VT and NVT, and more than one NVT). VT, vaccine serotypes/serogroups (4, 6A/B, 9A/L/N/V, 18A/B/C, 19B/F, and 23F). NVT, nonvaccine serotypes/serogroups (1, 3, 5, 6 C/D, 7C, 10A, 11A/B/C/D/F, 12A/B/F, 13, 15 A/B/C/F, 16F, 17F, 19A, 20, 21, 23A/B, and 34/37/17A).

b The number of children (percentage shown in parentheses) cocolonized who had not been vaccinated or had been vaccinated with 7-valent pneumococcal conjugate vaccine (PCV7).

c The *P*  values were determined by multivariate logistic regression, controlling for race, smoking household contact, co-trimoxazole use, day care attendance, and mean age at time of sample collection. *P* values of <0.05 were considered significant.

d Nontypeable, *lytA*-positive, serotype-negative samples.

Among children colonized with multiple serotypes, concurrent colonization by PCV7 serotypes and NVT was lower among PCV7-vaccinated children than in PCV-unvaccinated children at both 9 months (62.1% versus 66.7%; *P* = 0.006) and 16 months (55.3% versus 63.2%; *P* = 0.004) of age. Concurrent colonization of multiple PCV7 serotypes only was also lower in PCV7-vaccinated children than in PCV-unvaccinated children at 9 months (13.8% versus 30.7%; *P* = 0.002) and 16 months (2.6% versus 22.4%; *P* = 0.005) of age. In contrast, concurrent carriage of multiple NVT was higher in PCV7-vaccinated children than in PCV-unvaccinated children at 9 months (24.1% versus 2.6%; *P* = 0.015) and 16 months (42% versus 14.3%; *P* = 0.002) of age.

**FIG 3:**
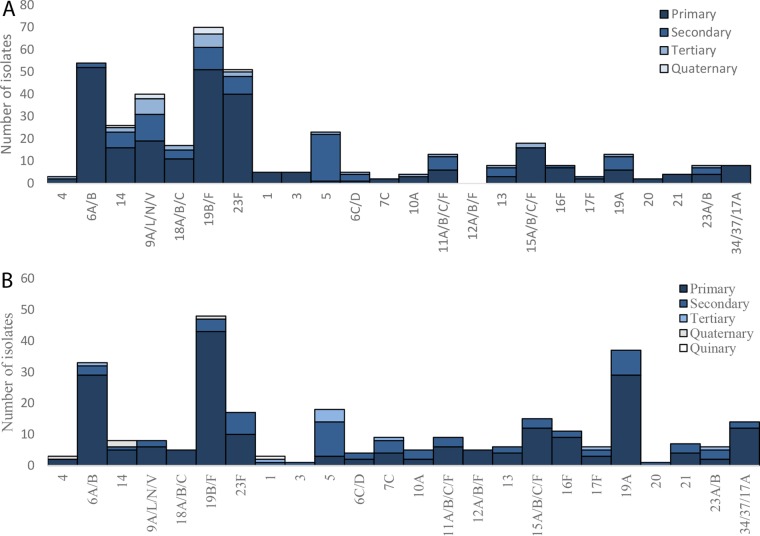
Serotype/serogroup-specific ranking of multiple pneumococcal carriage in PCV-unvaccinated children (A) and PCV7-vaccinated children (B). Each *S. pneumoniae* isolate was ranked according to its carriage density to those of other isolates present in the same sample as determined by quantitative PCR (qPCR). Single colonizers were included in the analysis as primary colonizing serotypes.

Among PCV-unvaccinated children, PCV7 serogroups/serotypes 6A/B (prevalence, 52/264, or 19.7%), 19B/F (prevalence, 51/264, or 19.3%), and 23F (prevalence, 40/265, or 15.2%) were the highest-density-colonizing serotypes. Furthermore, nonvaccine serotype 5 was the most prevalent second colonizing serotype, ranked by having a lower density than the primary colonizing serotype but a higher density than the tertiary colonizing serotype. PCV7 serogroup 9A/L/N/V was the most prevalent tertiary colonizing serotype/serogroup ranked by having a lower density than both the primary and secondary colonizing serotypes (Fig. 3A). Among PCV7-vaccinated children, PCV7 serogroups 19B/F (14.7%) and 6A/B (14.7%) and nonvaccine serotype 19A (14.7%) were the highest-density-colonizing serotypes, while nonvaccine serotype 5 was the most common second (16.2%) and third (44.4%) colonizing serotype based on its density of carriage (Fig. 3B).

**FIG 4:**
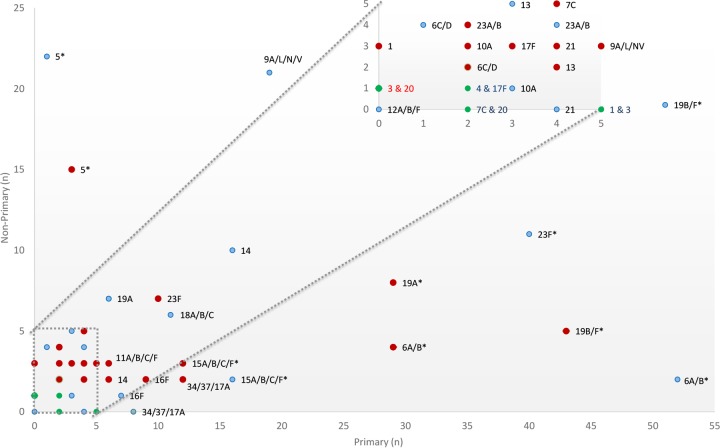
Serotype-specific propensity of whether a given serotype/serogroup is more likely to be found as a primary or nonprimary isolate in PCV7-vaccinated (red) and PCV-unvaccinated (blue) children. Pneumococcal serotypes identified from nasopharyngeal swabs were classified according to whether they occurred as a primary or nonprimary isolate as determined by carriage density. Single colonizers were included in the analysis as primary isolates. The box at the top of the graph is an expanded view of the block in the lower left-hand corner of the graph. Green dots represent two or more serotypes with the same colonizing profile. Results were considered significant when the *P* value was <0.05, and these results are indicated by an asterisk.

The serotype-specific propensity of whether a given serotype/serogroup is more likely to be found as a primary or nonprimary isolate is shown in Fig. 4, with vaccine serogroups 6A/B and 19B/F and nonvaccine serotypes/groups 15A/B/C/F and 34/37/17A being more likely to be identified as primary isolates than nonprimary isolates in both PCV7-vaccinated and PCV-unvaccinated children. Nonvaccine serotype 19A was, however, more likely identified as a primary isolate than as a nonprimary isolate in PCV7-vaccinated children only. Further, nonvaccine serotype 5 was more likely identified as a nonprimary isolate in both PCV7-vaccinated and PCV-unvaccinated groups. These results did not differ between study time points.

**FIG 5:**
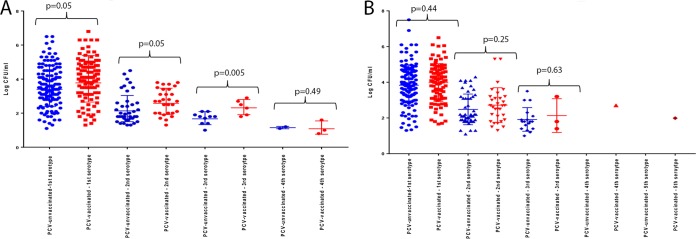
Density of concurrent colonizing pneumococcal serotypes between PCV7-vaccinated and PCV-unvaccinated children who were 9 months old (A) and 16 months old (B), grouped by first, second, third, and fourth colonizing serotype as determined by density of carriage.

PCV7-vaccinated children compared to PCV-unvaccinated children at 9 months of age had a higher mean density of colonization for the first (3.9 versus 4.38 CFU/ml; *P* = 0.05), second (3.59 versus 3.99 CFU/ml; *P* = 0.05) and third (1.7 versus 2.35 CFU/ml; *P* = 0.005) colonizing serotypes; however, no difference in densities were found by the time the children were 16 months old (Fig. 5).

## DISCUSSION

In this study, quantitative PCR was used to compare pneumococcal serotype/serogroup-specific colonization in cohorts of PCV7-vaccinated and PCV-unvaccinated African children. We clearly showed the vaccine effect, typified by a decrease in the prevalence of PCV7 serotype colonization and a corresponding increase in NVT colonization (5–7); this study also showed that both mechanisms of serotype replacement and unmasking led to the increase in NVT colonization in PCV7-vaccinated children. Some serotypes were associated with an absolute increase in colonization (replacement), as both the colonization prevalence and density increased and were commonly found as primary colonizers in PCV-vaccinated children while they were found equally as primary and nonprimary colonizers in PCV-unvaccinated children. Other serotypes were associated with an increase in detection (unmasking), as only the colonization density increased and they were commonly found as second and third colonizers in both PCV-vaccinated and PCV-unvaccinated children.

Although serotype-specific analysis was often limited by a small sample size for a given serotype, the carriage prevalence of most NVT remained unchanged in PCV7-vaccinated children, with the exception of serotype 19A, for which the prevalence of colonization was higher. Although this difference in serotype 19A has been previously documented in PCV7-vaccinated populations (5, 8–10), the qPCR method allowed quantification and showed a higher density of this serotype. Also, serotype 19A was more commonly reported as a primary isolate in PCV7-vaccinated children, while being equally a primary (46.2%) or nonprimary (53.8%) isolate in PCV-unvaccinated children. This would indicate that the difference in serotype 19A carriage among PCV7-vaccinated children was due to a combination of serotype replacement and unmasking of colonization which would have been missed using conventional culture methods. In addition, the higher carriage prevalence and density of serotype 19A has clinical relevance, as it could explain the emergence of this serotype as the major replacement serotype causing invasive pneumococcal disease (IPD) following PCV7 introduction in several settings, most likely as this serotype commonly has a high prevalence of antimicrobial resistance clones, thus facilitating its survival compared to antibiotic-susceptible NVT (11–13).

The higher colonization density of nonvaccine serotype 5 and serogroup 23A/B was identified in PCV7-vaccinated children compared to PCV-unvaccinated children in the absence of a difference in overall colonization prevalence. As serotype 5 is rarely seen in conventional carriage studies, these results suggest an unmasking of serotype 5 by the qPCR method. Nonetheless, serotype 5 has a high invasive disease potential in our setting (14, 15), and a higher carriage density as a result of PCV immunization could enhance its ability to cause mucosal and invasive disease that could potentially offset the effectiveness of PCV7 immunization. Further, a composition shift in cocolonized children from a mixture of NVT and vaccine serotypes to almost pure NVT as a result of PCV7 immunization was observed, supporting an unmasking of serotype carriage. Children with multiple serotype colonization, however, also had a lower density of colonization with NVT than children colonized with only one serotype. These observations therefore further support increases in detection probability (unmasking) and NVT acquisition (true replacement) to explain the higher rate of NVT colonization among PCV7-vaccinated children (2).

PCV7 was introduced into the public immunization program in May 2009 in South Africa and replaced by PCV13, which includes serotypes 19A and 5, in April 2011. These serotypes that have a high invasive disease potential in our settings, were thus considered nonvaccine serotypes at the time of our studies. Further, qPCR increases the detection of serotypes 5 and 19A previously missed by traditional culture methods (4) and highlights the importance of continued surveillance using sensitive molecular methods able to detect serotypes at a low carriage density to more accurately measure the effectiveness of these vaccines in reducing/eliminating carriage or change in the circulating pneumococcal serotypes. For example, after the introduction of PCV13, serotype 12F has emerged as a leading “replacement” serotype, causing IPD in many settings (16–18).

Of note among PCV7-vaccinated children, despite a lower prevalence of PCV7 serotype colonization at 9 months of age, the density of colonization of both PCV7 serotype and NVT pneumococci was higher than in PCV-unvaccinated children. Although we might expect PCV vaccination to reduce the density of vaccine serotype carriage, the opposite was found. One explanation for this observation is that pneumococcal carriage density may be influenced by antibodies to common pneumococcal surface antigens (CPAs). Ditse et al. (19) found lower titers to CPAs in PCV7-vaccinated infants than in PCV-unvaccinated infants at 10 months of age, most likely from reduced pneumococcal exposure. By the time the infants were 18 months old, however, these differences were not significant (19), which was consistent with colonization density noted in our study. Another explanation could be that PCV is less effective in preventing colonization with PCV7 serotypes among a subset of children who remained colonized, possibly due to poorer immune response or other factors contributing to the colonization by these PCV7 serotypes, which also enables a higher density of colonization by these serotypes.

Although we have shown the value of molecular assays for surveillance of pneumococcal colonization, limitations of our study include the fact that the qPCR assays could not discriminate between all vaccine serotypes within their respective serogroups due to the high genotypic similarities within each group as described previously (4); however, due to the high concordance between serotypes identified by culture and qPCR, we can assume from colonization data using traditional culture methods that 93.3%, 88.9%, and 91.4% of all 9A/L/N/V, 18A/B/C, and 19B/F serogroups identified by qPCR to be vaccine serotypes 9V, 18C, and 19F, respectively. The qPCR assay did not detect all pneumococcal serotypes and nontypeable pneumococci could not be identified. This may have limited our understanding of the roles of nontypeable and other serotypes in limiting vaccine effect for protection against colonization, especially since recent genome sequencing projects have shown an increase in nontypeable isolates following PCV immunization (20). Further, this study did not have adequate power to evaluate serotype-specific differences. Another limitation is that the control group was enrolled after the vaccinated group; however, the limited coverage of PCV in the community at the time made it unlikely that a broader PCV7 indirect effect reduced transmission by young vaccinated children to unvaccinated children in the community. Last, as the study was not a randomized clinical trial and the cohorts were not matched, unmeasured factors could have influenced the results.

In conclusion, molecular qPCR allowed us to gain a better understanding of serotype carriage and indicated that the underlying mechanisms for the increase in NVT colonization in PCV7-vaccinated children was likely due to both serotype replacement and unmasking of underlying preexisting colonizing serotypes which were previously undetected by conventional culture methods.

## MATERIALS AND METHODS

### Study population.

Archived nasopharyngeal swab samples collected from pneumococcal conjugate vaccine (PCV)-unvaccinated and 7-valent PCV (PCV7)-vaccinated cohorts of HIV-uninfected children from Soweto, South Africa, were retrospectively analyzed. Detailed information of the study cohorts has been described previously (21, 22). Briefly, the PCV7-vaccinated cohort was enrolled between April 2005 and June 2006 and included 125 HIV-exposed-uninfected (HEU) infants born to HIV-infected mothers and 125 HIV-unexposed infants, with all infants between 6 and 12 weeks old at enrollment. These infants received three doses of PCV7 (Prevnar; Wyeth Vaccines, NJ, USA) at 6, 10, and 14 weeks of age (22, 23). From January 2007 through October 2007, 251 PCV7-naïve infants, including 125 HEU infants and 126 HIV-unexposed infants were also enrolled in a separate pneumococcal carriage study (21). During both studies, pneumococcal immunization of children in Soweto, South Africa (birth cohort of approximately 28,000 per annum) was limited mainly to study participants (approximately 600 children in total participated in PCV studies at that time), as PCV7 was introduced into the pubic immunization program in May 2009 (24).

Nasopharyngeal swabs were collected from participants in both cohorts at several time points, including at 9 and 15 to 16 months of age. Swabs were stored in skim milk-tryptone-glucose-glycerol (STGG) transport medium at the Respiratory and Meningeal Pathogen Research Unit (RMPRU) in South Africa, as recommended by WHO (25). The samples had been previously cultured for *Streptococcus pneumoniae* using standard culture methods, and pneumococcal serotyping was undertaken using the Quellung method as described previously (21). Direct comparison of pneumococcal serotype colonization between the PCV7-vaccinated and PCV-unvaccinated cohorts was not performed.

### Multiplex quantitative PCR methods.

Briefly, stored nasopharyngeal swabs were thawed, and total nucleic acids were extracted using the automated NucliSens easyMAG extraction system (BioMérieux, Marcy l’Etoile, France) according to the manufacturer’s instructions; extracted nucleic acids were stored at −20°C. The quantitative PCR (qPCR) method used in this study has previously been described and validated (4). Briefly, target DNAs were prescreened for the *Streptococcus lytA* gene (26). All samples with quantification cycle (Cq) values of <35 were regarded as positive for streptococci and further molecularly serotyped for PCV7 serotypes/serogroups (4, 6A/B, 9A/L/N/V, 14, 18A/B/C, 19B/F, and 23F) and nonvaccine serotypes/serogroups (1, 3, 4, 5, 6C/D, 10A, 11A/B/C/D/F, 12A/B/F, 13, 15A/B/C/F, 16F, 17F, 19A, 20, 21, 23A/B, and 34/37/17 A). Amplification data were analyzed with the Applied Biosystems 7500 software, version 2.3 (Foster City, CA, USA) with manually defined thresholds. Negative samples were defined as those with Cq values of ≥35. Further, all *lytA*-negative samples were tested for the human glyceraldehyde-3-phosphate dehydrogenase (GAPDH) target to confirm the efficiency of the DNA extraction, with all qPCR *lytA*-negative samples being positive for GAPDH.

### Statistical analysis.

The Pearson χ^2^ test or Student *t* test was used to compare baseline characteristics between the vaccinated and unvaccinated cohorts. Comparisons of prevalence of pneumococcal colonization between cohorts were analyzed using multiple logistic regression models adjusted for race, passive smoke exposure, day care attendance, co-trimoxazole usage, and mean age at sample collection; adjusted odd ratios (aOR) were calculated. Colonization density data were presented as CFU/milliliter and geometric mean densities (GMD). Confidence intervals (95% confidence intervals [95% CI]) of pneumococcal concentrations were calculated following log_10_ transformation, using analysis of covariance adjusted for possible covariates. Serotype-specific propensity comparing the observed proportion of a given serotype/serogroup found as a primary or nonprimary isolate were analyzed using a two-tail binomial test, in which the primary isolate was defined as the first dominant colonizing serotype that had the highest colonization density among other cocolonizing serotypes identified in the same sample, or was a single colonizer. Nonprimary isolates were defined by having a lower carriage density than the primary isolates. Results were considered significant when the *P* values were <0.05. Statistical analysis was performed with Stata version 11.0 (Statacorp, TX, USA).

### Ethics.

Ethical approval for the original two studies was obtained from the Medical Human Research Ethics Committee (HREC) of the University of the Witwatersrand {vaccinated cohort [HREC (040704)], and Clinical trials registration number NCT00099658; PCV-unvaccinated cohort [HREC (050705)]}. Approval for further testing of samples was obtained from the HREC (M120972). Written, informed consent was obtained from the parents/guardians of the study participants at the time of enrollment.

### Data availability.

The data that support the findings of this study are available from the corresponding author upon request.

## References

[1] Robinson J 2004 Colonization and infection of the respiratory tract: what do we know? Paediatr Child Health 9:21–24. doi:10.1093/pch/9.1.21. 19654976 PMC2719511

[2] Weinberger DM, Malley R, Lipsitch M 2011 Serotype replacement in disease after pneumococcal vaccination. Lancet 378:1962–1973. doi:10.1016/S0140-6736(10)62225-8. 21492929 PMC3256741

[3] Lipsitch M, Dykes JK, Johnson SE, Ades EW, King J, Briles DE, Carlone GM 2000 Competition among Streptococcus pneumoniae for intranasal colonization in a mouse model. Vaccine 18:2895–2901. doi:10.1016/S0264-410X(00)00046-3. 10812233

[4] Olwagen CP, Adrian PV, Madhi SA 2017 Comparison of traditional culture and molecular qPCR for detection of simultaneous carriage of multiple pneumococcal serotypes in African children. Sci Rep 7:4628. doi:10.1038/s41598-017-04915-y. 28680083 PMC5498530

[5] Cheung YB, Zaman SM, Nsekpong ED, Van Beneden CA, Adegbola RA, Greenwood B, Cutts FT 2009 Nasopharyngeal carriage of Streptococcus pneumoniae in Gambian children who participated in a 9-valent pneumococcal conjugate vaccine trial and in their younger siblings. Pediatr Infect Dis J 28:990–995. doi:10.1097/INF.0b013e3181a78185. 19536041

[6] Spijkerman J, van Gils EJ, Veenhoven RH, Hak E, Yzerman EP, van der Ende A, Wijmenga-Monsuur AJ, van den Dobbelsteen GP, Sanders EA 2011 Carriage of Streptococcus pneumoniae 3 years after start of vaccination program, the Netherlands. Emerg Infect Dis 17:584–591. doi:10.3201/eid1704.101115. 21470445 PMC3377405

[7] Huang SS, Platt R, Rifas-Shiman SL, Pelton SI, Goldmann D, Finkelstein JA 2005 Post-PCV7 changes in colonizing pneumococcal serotypes in 16 Massachusetts communities, 2001 and 2004. Pediatrics 116:e408–e413. doi:10.1542/peds.2004-2338. 16140686

[8] Duggan ST 2010 Pneumococcal polysaccharide conjugate vaccine (13-valent, adsorbed) [Prevenar 13^R^]. Drugs 70:1973–1986. doi:10.2165/11205110-000000000-00000. 22149553

[9] Huang SS, Hinrichsen VL, Stevenson AE, Rifas-Shiman SL, Kleinman K, Pelton SI, Lipsitch M, Hanage WP, Lee GM, Finkelstein JA 2009 Continued impact of pneumococcal conjugate vaccine on carriage in young children. Pediatrics 124:e1–e11. doi:10.1542/peds.2008-3099. 19564254 PMC2782668

[10] Hanquet G, Kissling E, Fenoll A, George R, Lepoutre A, Lernout T, Tarragó D, Varon E, Verhaegen J 2010 Pneumococcal serotypes in children in 4 European countries. Emerg Infect Dis 16:1428–1439. doi:10.3201/eid1609.100102. 20735928 PMC3294971

[11] Pelton SI, Huot H, Finkelstein JA, Bishop CJ, Hsu KK, Kellenberg J, Huang SS, Goldstein R, Hanage WP 2007 Emergence of 19A as virulent and multidrug resistant Pneumococcus in Massachusetts following universal immunization of infants with pneumococcal conjugate vaccine. Pediatr Infect Dis J 26:468–472. doi:10.1097/INF.0b013e31803df9ca. 17529860

[12] Vestrheim DF, Steinbakk M, Aaberge IS, Caugant DA 2012 Postvaccination increase in serotype 19A pneumococcal disease in Norway is driven by expansion of penicillin-susceptible strains of the ST199 complex. Clin Vaccine Immunol 19:443–445. doi:10.1128/CVI.05563-11. 22237889 PMC3294600

[13] Messina AF, Katz-Gaynor K, Barton T, Ahmad N, Ghaffar F, Rasko D, McCracken GH Jr 2007 Impact of the pneumococcal conjugate vaccine on serotype distribution and antimicrobial resistance of invasive Streptococcus pneumoniae isolates in Dallas, TX, children from 1999 through 2005. Pediatr Infect Dis J 26:461–467. doi:10.1097/INF.0b013e31805cdbeb. 17529859

[14] Hausdorff WP, Siber G, Paradiso PR 2001 Geographical differences in invasive pneumococcal disease rates and serotype frequency in young children. Lancet 357:950–952. doi:10.1016/S0140-6736(00)04222-7. 11289365

[15] von Gottberg A, Cohen C, de Gouveia L, Meiring S, Quan V, Whitelaw A, Crowther-Gibson P, Madhi SA, Whitney CG, Klugman KP 2013 Epidemiology of invasive pneumococcal disease in the pre-conjugate vaccine era: South Africa, 2003-2008. Vaccine 31:4200–4208. doi:10.1016/j.vaccine.2013.04.077. 23684826

[16] Zulz T, Wenger JD, Rudolph K, Robinson DA, Rakov AV, Bruden D, Singleton RJ, Bruce MG, Hennessy TW 2013 Molecular characterization of Streptococcus pneumoniae serotype 12F isolates associated with rural community outbreaks in Alaska. J Clin Microbiol 51:1402–1407. doi:10.1128/JCM.02880-12. 23408692 PMC3647894

[17] Chaguza C, Cornick JE, Andam CP, Gladstone RA, Alaerts M, Musicha P, Peno C, Bar-Zeev N, Kamng’ona AW, Kiran AM, Msefula CL, McGee L, Breiman RF, Kadioglu A, French N, Heyderman RS, Hanage WP, Bentley SD, Everett DB 2017 Population genetic structure, antibiotic resistance, capsule switching and evolution of invasive pneumococci before conjugate vaccination in Malawi. Vaccine 35:4594–4602. doi:10.1016/j.vaccine.2017.07.009. 28711389 PMC5571440

[18] Janoir C, Lepoutre A, Gutmann L, Varon E (ed) 2016 Insight into resistance phenotypes of emergent non 13-valent pneumococcal conjugate vaccine type pneumococci isolated from invasive disease after 13-valent pneumococcal conjugate vaccine implementation in France. Open Forum Infect Dis 3:ofw020. doi:10.1093/ofid/ofw020. 26955644 PMC4777900

[19] Ditse Z, Adrian PV, Kuwanda L, Madhi SA 2013 Association of Streptococcus pneumoniae common protein antigen (CPA) antibodies and pneumococcal nasopharyngeal colonization in HIV-infected and HIV-uninfected African children. Vaccine 31:4421–4427. doi:10.1016/j.vaccine.2013.06.097. 23845819

[20] Roca A, Bojang A, Bottomley C, Gladstone RA, Adetifa JU, Egere U, Burr S, Antonio M, Bentley S, Kampmann B, Pneumo13 Study Group 2015 Effect on nasopharyngeal pneumococcal carriage of replacing PCV7 with PCV13 in the Expanded Programme of Immunization in The Gambia. Vaccine 33:7144–7151. doi:10.1016/j.vaccine.2015.11.012. 26592141 PMC5352730

[21] Nunes MC, Shiri T, van Niekerk N, Cutland CL, Groome MJ, Koen A, von Gottberg A, de Gouveia L, Klugman KP, Adrian PV, Madhi SA 2013 Acquisition of Streptococcus pneumoniae in pneumococcal conjugate vaccine-naive South African children and their mothers. Pediatr Infect Dis J 32:e192–e205. doi:10.1097/INF.0b013e31828683a3. 23340555

[22] Madhi SA, Adrian P, Cotton MF, McIntyre JA, Jean-Philippe P, Meadows S, Nachman S, Käyhty H, Klugman KP, Violari A, Comprehensive International Program of Research on AIDS 4 Study Team 2010 Effect of HIV infection status and anti-retroviral treatment on quantitative and qualitative antibody responses to pneumococcal conjugate vaccine in infants. J Infect Dis 202:355–361. doi:10.1086/653704. 20583920 PMC2902789

[23] Madhi SA, Izu A, Nunes MC, Violari A, Cotton MF, Jean-Philippe P, Klugman KP, von Gottberg A, van Niekerk N, Adrian PV, CIPRA 4 team 2015 Longitudinal study on Streptococcus pneumoniae, Haemophilus influenzae and Staphylococcus aureus nasopharyngeal colonization in HIV-infected and -uninfected infants vaccinated with pneumococcal conjugate vaccine. Vaccine 33:2662–2669. doi:10.1016/j.vaccine.2015.04.024. 25910923 PMC4436702

[24] Madhi SA, Bamford L, Ngcobo N 2014 Effectiveness of pneumococcal conjugate vaccine and rotavirus vaccine introduction into the South African public immunisation programme. S Afr Med J 104:228–234. doi:10.7196/SAMJ.7597. 24893498

[25] O’Brien KL, Bronsdon MA, Dagan R, Yagupsky P, Janco J, Elliott J, Whitney CG, Yang YH, Robinson LG, Schwartz B, Carlone GM 2001 Evaluation of a medium (STGG) for transport and optimal recovery of Streptococcus pneumoniae from nasopharyngeal secretions collected during field studies. J Clin Microbiol 39:1021–1024. doi:10.1128/JCM.39.3.1021-1024.2001. 11230421 PMC87867

[26] Carvalho MDG, Tondella ML, McCaustland K, Weidlich L, McGee L, Mayer LW, Steigerwalt A, Whaley M, Facklam RR, Fields B, Carlone G, Ades EW, Dagan R, Sampson JS 2007 Evaluation and improvement of real-time PCR assays targeting lytA, ply, and psaA genes for detection of pneumococcal DNA. J Clin Microbiol 45:2460–2466. doi:10.1128/JCM.02498-06. 17537936 PMC1951257

